# Serum KL-6 as a biomarker to predict progression at one year in interstitial lung disease

**DOI:** 10.1038/s41598-025-22483-4

**Published:** 2025-10-09

**Authors:** Francesco Bonella, M. C. Vegas Sanchez, M. d’Alessandro, P. Millan-Billi, R. F. Santos, N. Schröder, H. N. Bastos, M. Molina-Molina, O. Sánchez Pernaute, D. Castillo Villegas, E. Bargagli

**Affiliations:** 1 Center for Interstitial and Rare Lung Disease, Pneumology Department, Ruhrlandklinik University Hospital, University of Duisburg-Essen, Essen, Germany; 2 Immunology Department, Jiménez Díaz Foundation University Hospital, Autonomous University of Madrid, Madrid, Spain; 3 Respiratory Diseases and Lung Transplant Unit, University of Siena, Siena, Italy; 4 Department of Respiratory Medicine, Hospital de la Santa Creu i Sant Pau, Barcelona, Spain; 5 i3S –Institute for Research and Innovation in Health, University of Porto, Porto, Portugal; 6 Faculty of Medicine, RISE – Health Research Network, University of Porto, University Hospital Center of São João, Porto, Portugal; 7 ILD Unit, Pulmonology Service, Bellvitge University Hospital - IDIBELL-Barcelona, CIBERES Bellvitge Biomedical Research Institute, Barcelona, Spain; 8 Rheumatology Department, Jiménez Díaz Foundation University Hospital, Autonomous University of Madrid, Madrid, Spain; 9 European Reference Network (ERN)-LUNG, ILD and SARC Core Network, Frankfurt, Germany

**Keywords:** Progressive phenotype, ILD, Serum KL-6, Biomarker, Predictive markers, Prognostic markers, Risk factors

## Abstract

About one third of non-IPF ILD patients progresses over time. Serum KL-6, a lung epithelial mucin type 1, is an established marker to assess disease severity in ILD but its ability to predict progression needs to be further explored. To investigate whether serum KL-6 is of additional value to stratify the patients for the risk of developing clinical or functional progression at one year. ILD patients from 6 European centers were retrospectively enrolled. Disease progression was defined as relative decline ⩾10% in FVC or ⩾15% in DLco from baseline. Serum KL-6 was measured using a full-automated chemiluminescent immunoassay (Fujirebio). Comparative logistic regression was used to identify predictors of progression at one year. 303 patients were included. 37% developed progression after one year from KL-6 measurement. A stepwise selection was used to identify and include five predictors of progression in a risk score: age, gender, BMI, FVC, and KL-6. The final model was superior to KL-6 alone to predict progression at one year, with 55% sensitivity, 73% specificity and 67% accuracy at a cut-off of 5. Patients were stratified in low and high risk of progression at one year based on the cut-off of 5, with a similar accuracy for IIP 0.687 and CTD-ILD 0.720 but not for HP. Serum KL-6 levels, included in a risk score with other clinical and functional variables, may help to better stratify patients for the risk of disease progression at one year, compared to any individual predictor.

**Supplementary Information:**

The online version contains supplementary material available at 10.1038/s41598-025-22483-4.

## Introduction

Interstitial lung disease (ILD) is a heterogeneous group of diseases characterized by impairment of respiratory function and unpredictable outcome^1–3^. Disease progression develops always over time in patients with idiopathic pulmonary fibrosis (IPF) and in 18–32% of those with other fibrotic ILDs^4^. There is no standard definition of disease progression in ILD, but it generally relies on lung function tests, symptoms and radiology^5^. The validation of non-invasive biomarkers for predicting disease progression, still a major unmet need in ILD, would be of practical help to identify those patients which may require early or more aggressive treatment or evaluation for lung transplant.

Clinical scores like Gender, Age and Physiology (GAP) have been used to stratify patients for the risk of mortality but their association with progression risk has been poorly investigated^6,7^.

Among all circulating proteins investigated in ILD, Krebs von den Lungen-6 (KL-6), a human MUC-1 mucin produced by regenerating pneumocytes type II, has been validated as a biomarker of disease activity in ILD mainly in Japan, where is used in the clinical routine^8–10^. Serum KL-6 levels have been found to reflect disease severity in fibrotic ILDs, especially those with underlying systemic autoimmune diseases, and higher levels can be predictive of acute exacerbation^11,12^.

Aim of our study was to verify whether serum KL-6 at baseline, alone or combined in a weighted clinical score, could improve stratification of ILD patients for risk of disease progression at one year.

## Patients and methods

### Study population and design

Patients with ILD followed up at 6 Institutions from four different countries were included in this retrospective cross-sectional analysis. Diagnosis of ILD was revised according to ATS/ERS criteria 2018 and 2013 and confirmed by the local ILD Board^13,14^. The study was approved by the lead local Institutional Review Board (IRB) (Essen, nr. 06-3170 and 10-4397; Barcelona (St Pau) 21/026 R-OBS; Barcelona (Bellvitge) PR033/21; Madrid PIC115-20_FJD; Siena C.E.A.V.S.E. nr. 17431; Porto CES 72 − 12) and all the subjects provided written informed consent. All methods were performed in accordance with the relevant guidelines and regulations.

### Measurements and definitions

Measurements of FVC and diffusing capacity of the lung for carbon monoxide (DLco) were performed at each institution at the time of serum KL-6 measurement. Pulmonary function test (PFT) results were expressed as percentages of predicted normal values (% pred.)^15^.

GAP score and stage were calculated as previously described^6^.

HRCT was performed in all patients at diagnosis. Fibrosis score was obtained by visual assessment performed by the local radiologists without using specific software or central review.

Serum samples were obtained in all subjects within 6 months after ILD diagnosis. On average, KL-6 was obtained 3±1.2 months after baseline HRCT. The samples were stored at -80 °C until analysis. Serum KL-6 was measured using the same automated chemiluminescent immunoassay (CLEIA) (Fujirebio Inc.).

Disease progression was defined as relative decline of ≥ 10% in FVC or ≥ 15% in DLco % between baseline measurements and the end-point 12 ± 3 months. To avoid subjective interpretation, radiology changes or symptoms were not considered for defining disease progression.

### Statistical analysis

Continuous variables were evaluated for a normal distribution with the Kolmogorov-Smirnov test. Parametric data are presented as mean ± standard deviation (SD) and non-parametric data as medians with interquartile ranges (IQR). Categorical variables are presented as either a percentage of the total, or numerically, as appropriate. Comparisons between the groups were evaluated using a two-tailed t-test, Mann-Whitney *U* or Kruskal-Wallis tests as appropriate for continuous variables, and Chi-squared or Fischer’s exact tests for categorical variables. The primary outcome was the progression at 1 year. Multivariate logistic regression analysis was performed to identify predictors of disease progression using the clinical variables at baseline (age, gender, BMI, serum KL-6, FVC% pred, DLco% pred, underlying ILD, fibrosis score, and presence of emphysema, dyspnea) as potential explanatory variables [Supplement Appendix A]. Each selected variable was assigned a weight proportional to its odds ratio (OR). Points were attributed according to the OR (OR 1.0–1.2 = 1; 1.2–1.4 = 2; 1.4–1.6 = 3). The total score was defined as the sum of the points. Subsequently, subjects were clustered in a high risk (HR) group versus a low risk (LR) group using the optimal threshold determined by a ROC analysis for disease progression at 1-year. Finally, contingency tables were constructed, and Chi-square test (p-value) was used to test the performance of the new risk score for disease progression.

P values of < 0.05 were considered statistically significant.

Sensitivity analyses were performed to corroborate primary results. Detailed methods and results with tables and figures are included in the Supplement Appendix B.

All statistical analyses were performed using Addinsoft (2022) XLSTAT statistical and data analysis solution (New York, USA).

## Results

### Characteristics of study subjects

We studied 303 ILD patients from 6 European centers. Of them, 131 (43%) had idiopathic interstitial pneumonia (IIP) (31 IPF and 100 fNSIP, fibrotic non-specific interstitial pneumonia), 82 (27%) had a form associated with systemic autoimmune disease (CTD) or with autoimmune features (IPAF), and 90 (30%) had hypersensitivity pneumonitis (HP, 82% of them having fibrotic HP). Demographic and laboratory characteristics of the subjects according to underlying ILD are shown in Table 1. The proportion of males, the presence of UIP pattern (typical/probable) and lung functional impairment were significantly different at baseline between ILDs.

**Table 1:** Demographics and characteristics of the studied subjects.

	All subjects	IIP	CTD/IPAF-ILD	HP	*p*
	*N* = 303	*N* = 131 (43%)	*N* = 82 (27%)	*N* = 90 (30%)	
Age, y median (IQR)	68.0 (60.8–73.8)	68.4 (60.7–75.3)	65.4 (59.3–72.3)	69.0 (63.0-72.4)	0.472
BMI, kg/m² median (IQR)	28.0 (25.6–31.0)	28.0(26.0- 30.5)	27.8 (24.5–31.9)	28.3 (26.0- 31.1)	0.685
Male gender, n (%)	182 (60.1)	102 (77.9)	31 (37.8)	49 (54.4)	< 0.0001
FVC, %pred median (IQR)	76.0 (64.0–90.0)	71.0 (59.7–83.1)	78.5 (66.3–89.3)	84.9 (69.8–97.0)	< 0.0001
DLco, %pred median (IQR)	54.0 (41.9–68.7)	45.5 (37.0–60.0)	57.1 (45.0-71.8)	63.0 (47.0-76.2)	< 0.0001
KL-6, U/mL median (IQR)	1287 (819–2177)	1351 (900–2195)	1208 (705–1878)	1254 (687–2395)	0.220
GAP stages, I/II/III (%)	60.4/33.8/5.8	45.4/43.8/10.8	70.7/29.3/0.0	74.1/22.2/3.7	< 0.0001
UIP pattern at HRCT, Yes / No (%)	48.8 / 51.2	67.9 / 32.1	26.8 / 73.2	34.0 / 66.0	< 0.0001
HRCT fibrosis score, </≥10% (%)	26.9 / 73.1	26.9 / 73.1	31.4 / 68.6	18.4 / 81.6	0.790
Emphysema, Yes (%)	43 (16.6)	28 (23.5)	6 (9.0)	9 (12.3)	0.065
Dyspnea, Yes (%)	222 (73.3)	93 (71.0)	64 (78.0)	65 (72.2)	0.113
Progressors, n (%)	111 (36.6)	52 (39.7)	25 (30.5)	34 (37.8)	0.384

Abbreviations: y = years; BMI = body mass index; PFT = lung function test; FVC = forced vital capacity; DLco = diffusing capacity for carbon monoxide; GAP = gender, age, physiology; HRCT = high resolution computed tomnography; ILD = interstitial lung disease; IIP = idiopathic interstitial pneumonia; CTD/IPAF-ILD = connective tissue disease or autoimmune feature-ILD; HP = hypersensitivity pneumonitis; KL-6 = Krebs von den Lungen-6; IQR = interquartile range; n = number.

### Disease progression

111 patients (37%) developed disease progression at one year from KL-6 measurement. Rate of progressors was similar across ILD groups (*p* = 0.384). The median decline in FVC % pred. in one year was − 12% (IQR − 20 to -3%) for progressors and 4% (IQR − 2 to 11%) for those who remained non-progressors. Similarly, median decline in DLco % pred. was − 19% (IQR − 29 to -11%) in progressors vs. 1% (-6% to 12%) in non-progressors (*p* < 0.0001) (Table 2). The distribution of GAP stages, FVC % pred of DLco % pred were not significantly different at baseline between progressors and non-progressors patients. Only gender, KL-6 and UIP pattern were significant different at baseline (Table 2).

**Table 2:** Comparison of clinical characteristics between patients who progressed and those who remained stable at one year.

	All subjects	Progressors	Non-progressors	*p* value
	*N* = 303	*N* = 111 (37%)	*N* = 192 (63%)	
Age, y (IQR)	68.0 (60.8–73.8)	67.0 (61.2–74.4)	68.1 (59.9–73.0)	0.241
BMI, kg/m² (IQR)	28.0 (25.6–31.0)	27.5 (25.6–31.2)	28.1 (25.6–30.9)	0.845
Male gender, n (%)	182 (61.0)	75 (67.6)	107 (55.7)	**0.043**
KL-6, U/mL (IQR)	1287 (819–2177)	1411 (900–2328)	1255 (718–1877)	**0.046**
FVC, %pred (IQR)	76.0 (64.0–90.0)	73.0 (64.4–89.0)	78.0 (64.0-90.2)	0.790
DLCO, %pred (IQR)	54.0 (41.9–68.7)	55.3 (44.1–70.2)	54.0 (42.0–68.0)	0.448
GAP stages, I/II/III (%)	60.4/33.8/5.8	52.3/39.3/8.4	65.1/30.6/4.3	0.071
UIP pattern at HRCT, Yes/No (%)	48.8 / 51.2	57.9 / 42.1	43.6 / 56.4	**0.027**
HRCT fibrosis score, </≥10% (%)	28.3 / 71.7	24.1 / 75.9	30.8 / 69.2	0.221
Emphysema, Yes, n (%)	43 (16.6)	19 (19.0)	24 (15.1)	0.135
Dyspnea, Yes, n (%)	222 (73.3)	84 (75.7)	138 (71.9)	0.730
ILD categories, n (%)				0.384
CTD-ILD/IPAF (*n* = 82)	82 (27.1)	25 (22.5)	57 (29.7)	0.183
HP (*n* = 90)	90 (29.7)	34 (30.6)	56 (29.2)	0.795
IIP (incl. IPF) (*n* = 131)	131 (43.2)	52 (46.8)	79 (41.1)	0.339
FVC % pred decline, % (IQR)	0% (-8% to 7%)	-12% (-20% to -3%)	4% (-2% to 11%)	< 0.0001
DLco %pred decline, % (IQR)	-4% (-14% to 7%)	-19% (-29% to -11%)	1% (-6% to 12%)	< 0.0001

Criteria to define progression are specified in the text of the results.

Abbreviations: BMI = body mass index; y = years; PFT = lung function test; FVC = forced vital capacity; DLco = diffusing capacity for carbon monoxide; GAP = gender, age, physiology; ILD = interstitial lung disease; IIP = idiopathic interstitial pneumonia; CTD/IPAF-ILD = connective tissue disease or autoimmune feature-ILD; HP = hypersensitivity pneumonitis; HRCT = high resolution computed tomography; KL-6 = Krebs von den Lungen-6; IQR = interquartile range; n = number.

### Serum KL-6 levels at baseline

**Fig. 1:**
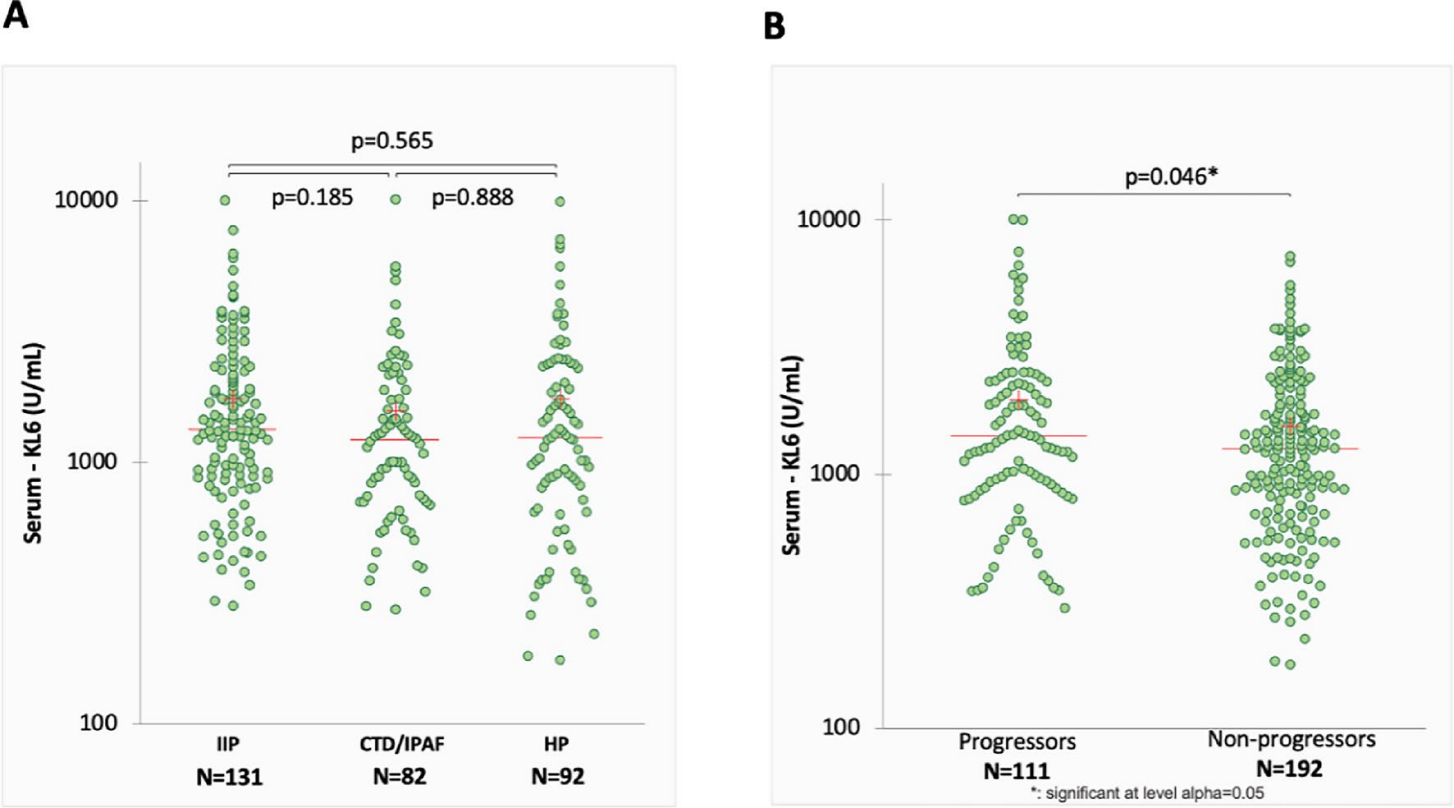
Distribution of serum KL-6 concentrations at baseline according to ILD type (**A**) and between progressors and non-progressors according to lung function decline definition at on year (disease progression at one year (**B**). Dots are single measurements; red cross represent mean values and red lines represent median values. Significance of the comparison is shown in the graphic.

Serum KL-6 concentration at baseline was 1287 (819–2177) U/ml in the entire cohort. No significant differences in baseline KL-6 concentrations according to the ILD disease group were seen (Table 1; Fig. 1A**).** Serum KL-6 levels at baseline tended to be higher in progressors vs. non progressors (*p* = 0.046) (Fig. 1B).

No correlation was observed between serum KL-6 levels and age, BMI, or link with gender. Serum KL-6 levels were inversely correlated with FVC pred (*r*= -0.149, *p* = 0.011) and DLco pred (*r*= -0.345, *p* < 0.0001) at baseline. The R²(adjusted) values of linear correlation between the PFT and the KL-6 measurements showed a weak dependence, 0.02 and 0.12 with FVC pred and DLco pred respectively. Baseline KL-6 levels did not significantly correlate with the % decline of FVC or DLco % pred. over one year.

### Multistep logistic regression for 1-year progression

In the multistep logistic regression analysis, we included first the selected qualitative variables. Only gender was significantly associated with progression at 1 year (Chi² 5.14, *p* = 0.023) [supplement Table 1]).

Then, we added gender to the logistic regression for continuous quantitative variables [supplement Table 2]. Using the quantitative variables plus gender in the logistic regression on all ILD groups, only KL-6 and gender showed a significant chi-square 6.58 (*p* = 0.010) and 4.50 (*p* = 0.034) respectively. We observed similar weights in IIP group but distinct weights for the model’s prediction in other ILD groups: highest chi² seen for BMI and FVC %pred in CTD/IPAF group and for Age in HP group. We maintained the selected quantitative variables to proceed forward and to prepare a scoring system based on discrete strata.

### Determination of variables strata by ROC analysis and contingency table

The strata of the quantitative variables to be included in the final score were determined by ROC analysis. The optimal thresholds, when similar across ILD groups, were finally selected based on to the highest specificity. The cut-offs were then assigned as follows: 1750 U/mL for KL-6 (Sp 73%, Se 41% and accuracy 61%); 70%pred for FVC (Sp 68%, Se 41% and accuracy 58%); 75 year old for age (Sp 82%, Se 23% and accuracy 61%) and 32 kg/m² for BMI (Sp 85%, Se 23% and accuracy 62%) [supplement Fig. 1]. A further cut-off for KL-6 was determined at 750 U/mL based on to the best sensitivity (Se 83%, Sp 27% and accuracy 48%). Contingency tables to adjust and verify the association with the progression at one year were obtained for each variable strata [supplement Fig. 2]. The final model’s prediction with the selected variable strata displayed a significant power to predict progression at 1 year (Chi²= 21.16 – *p* = 0.007) and a good fit (Hosmer–Lemeshow test probability > chi²=0.988) [supplement Table 3]. The logit formula based on standardized coefficients gave a ROC AUC of 0.651 with sensitivity 31%, specificity 89% [supplement Fig. 3].

**Fig. 2:**
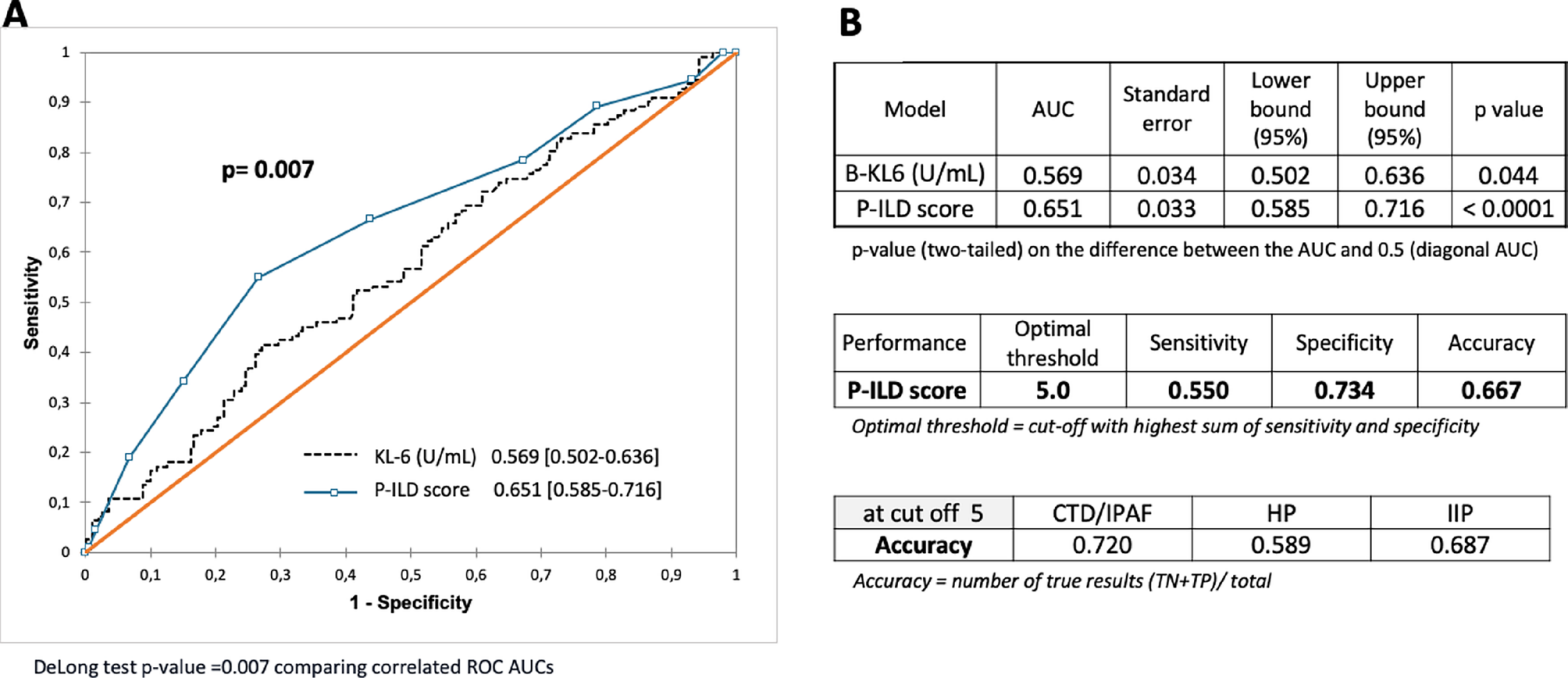
ROC curve for the P-ILD score vs. serum KL-6 alone (**A**) and performance of the P-ILD score (**B**) to predict progression at one year. Dots on the P-ILD score line represent the score points.

**Table 3:** Scoring model by using logistic regression weights.

Variable	β coeff.	Wald Chi-Square	Pr > Chi²	Wald Lower bound (95%)	Wald Upper bound (95%)	OR (expβ)	Points
Gender-F	0.000						
Gender-M	0.147	4.363	0.037	0.009	0.285	1.403	2
Age < 60	0.000						
Age < 60–75>	0.069	0.642	0.423	-0.099	0.237	1.172	1
Age > 75	0.145	2.953	0.086	-0.020	0.311	1.397	2
KL6 < 750	0.000						
KL6 < 750–1750>	0.035	0.139	0.709	-0.147	0.216	1.083	1
KL6 > 1750	0.185	4.180	0.041	0.008	0.362	1.530	3
FVC ≥ 70	0.000						
FVC < 70	0.105	2.283	0.131	-0.031	0.241	1.273	2
BMI < 22–32>	0.000						
BMI < 22	0.089	1.764	0.184	-0.042	0.221	1.228	1
BMI > 32	0.148	4.857	0.028	0.016	0.280	1.407	2

BMI = body mass index; y = years; FVC = forced vital capacity; KL6 = Krebs von den Lungen-6; OR = odds ratio; β = standardized coefficient from logistic regression; Ch² = chi-square.

**Fig. 3:**
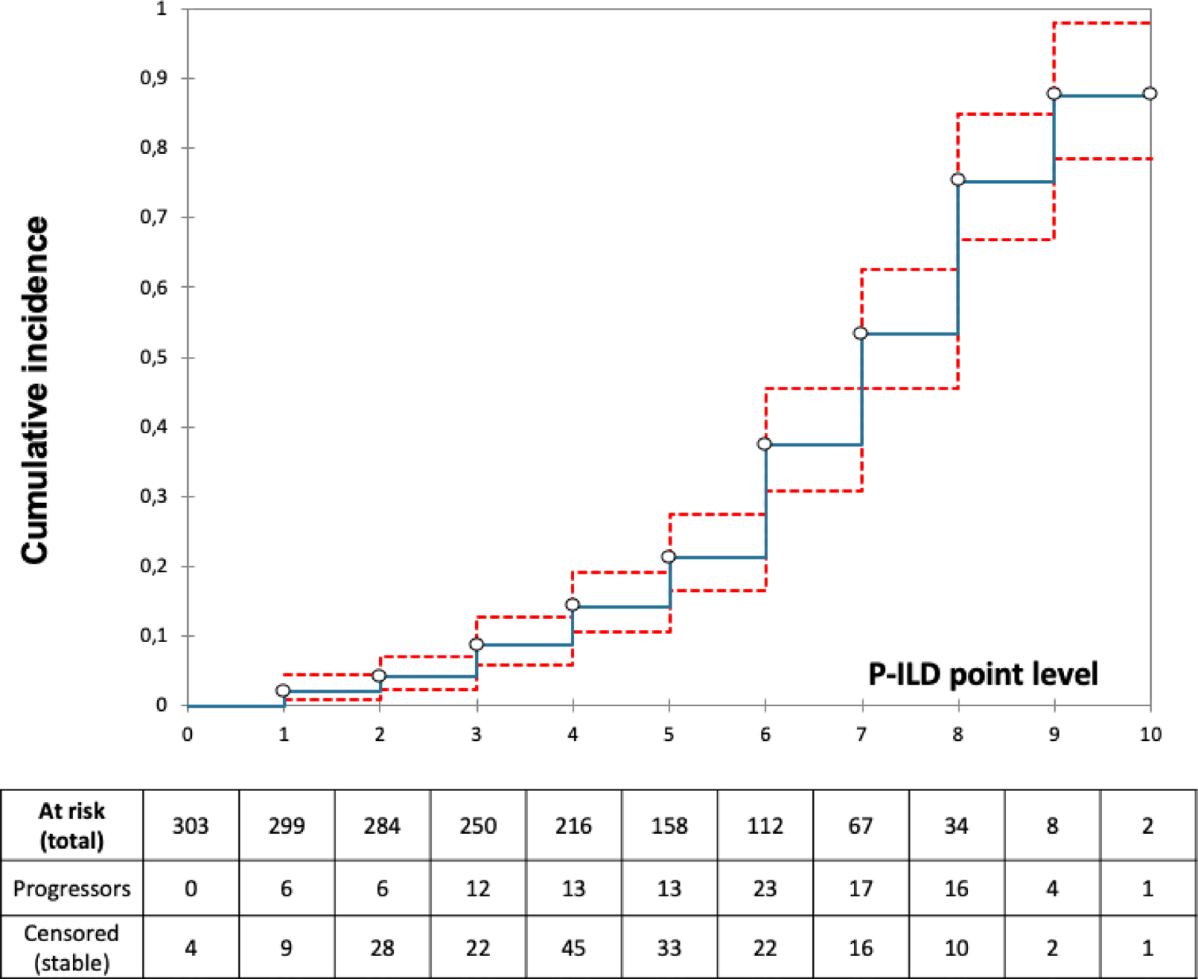
Cumulative incidence of progressors according to increasing P-ILD scores. Circles represent the cumulative incidence at each P-ILD point, red dotted line the 95% confidence interval. Total of patients at risk based on the P-ILD point level are shown under the graphic.

### A progression score to stratify patients at low / high risk for disease progression at one year

Based on the odds ratios from the logistic regression, points were assigned to each strata of the selected variables (gender, age, BMI, FVC and KL-6) to create a point-score model for disease progression at one year (P-ILD score), as shown in Table 3. The risk score for each patient was calculated by summing the points. The performance of the P-ILD score for predicting progression was examined by ROC analysis, and the AUC was 0.651 (95% CI 0.585–0.716, *P* < 0.0001) similar to the complex logit formula. The optimal cut-off value for P-ILD score was 5, as indicated by the maximum sum sensitivity + specificity. The sensitivity was 55%, specificity 73% and accuracy 67% overall [Fig. 2A and B]. Ultimately, the P-ILD score was divided in two risk levels: low risk (LR) for a score from 0 to equal 5 points and high risk (HR) for a score above 5 points.

**Fig. 4:**
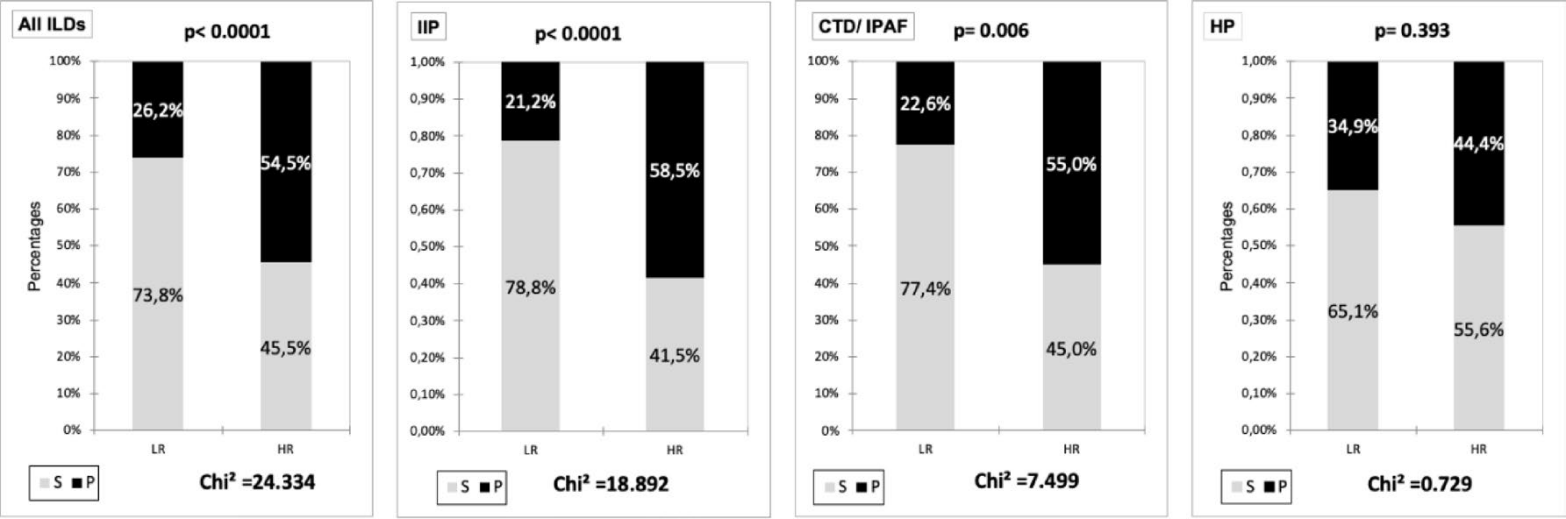
Contingency analysis for distribution of progressors (P) and non-progressors (S) in the low-risk (LR) and high-risk (HR) groups based on the P-ILD score > 5 points.

The cumulative incidence of progression at 1 year was closely linked to the cumulative P-ILD score points [Figure 3]. Contingency tables showed that the P-ILD score groups were overall associated with progression at 1year (Chi² = 24.3, *p* < 0.0001): 74% of LR group had stable ILDs whereas 55% of HR group had progression [Figure 4]. Among IIP patients, 79% of those in the P-ILD LR group remained stable and 59% of P-ILD HR group progressed at one year (overall Chi² = 18.9, *p* < 0.0001). Similarily, in the CTD/IPAF group, 77% of P-ILD LR patients remained stable and 55% of the P-ILD HR group had progression (Chi² = 7.5, *p* = 0.006). In the HP group, the performance of P-ILD score didn’t reach the statistical significance (*p* = 0.393).

Finally, likelihood-based measures demonstrated a superior performance of the P-ILD score to serum KL-6 alone, continuous or using strata with a Chi² of 21.2 (*p* = 0.007) versus 6.1 (*p* = 0.014) or 7.6 (*p* = 0.023) respectively. Supplement Table 4 summarized the baseline characteristic differences according to P-ILD risk score. The rate of progressors was 54.5% in P-ILD HR group versus 26.2% in LR group (*p* < 0.0001). All baseline characteristics of patients are significantly different between the two P-ILD score- based risk groups, except emphysema and dyspnea.

### Sensitivity analyses

In order to corroborate the results of the primary analysis, we performed several sensitivity analyses, which are shown in detail in the Supplement Appendix B. We could confirm that the P-ILD score works when different definitions of progression based on relative decline in FVC and Dlco are applied, but not when progressive pulmonary fibrosis (PPF) definition is used. PPF relies on absolute decline in FVC and DLco, therefore is less sensitive in identifying progression (but more precise). Therefore, definitions of progression relying on relative decline in FVC are usually included in clinical trials with 6–12 months of duration, otherwise with PPF the number of events would be too in relation to the trial population size. In fact, in our cohort we found that the absolute decline at 1 year in FVC and Dlco was influenced by the baseline values of the respective parameters, revealing a weakness of PPF criteria in this setting, and corroborating the use of the progression definition based on relative decline, as we did in the primary analysis.

In addition, we found a correlation between the identified KL-6 strata and baseline FVC and DLco at baseline, as well as with FVC decline at one year. This was not the case for Dlco decline, probably due to the high number of missing data at one year, which supported the exclusion of DLco from the P-ILD score.

Finally, we confirmed that the lack of performance of the P-ILD score for HP group also when other definitions of progression were applied.

## Discussion

In our study we found that serum KL-6 concentrations at baseline can be effectively used to identify ILD patients at risk of progression at one year. The P-ILD score, integrating clinical variables and baseline serum KL-6 concentrations, seems to have a better performance compared to any other predictor alone, for predicting disease progression at one year.

Serum KL-6 has been widely investigated as a biomarker for assessing disease severity in ILD, mainly in Japan^11,16–19^. It is known that serum KL-6 concentrations strongly correlate with FVC and DLco^20–22^ and rise proportionally to the extent of fibrosis on HRCT, whereas longitudinal data are lacking^23^. In our multicenter study, we could confirm the correlation of KL-6 with lung functional impairment at baseline, in line with previous reports. On the other hand, baseline serum KL-6 concentrations did not correlate with the magnitude of FVC or DLco changes over time, an issue, which has also been raised by other retrospective studies^24–26^. Longitudinal studies investigating changes of KL-6 serum concentrations from baseline demonstrated a better correlation with FVC or DLco decline at the same time point than at baseline^27,28^.

The primary aim of our study was to assess the role of KL-6 and further clinical variables as predictors of lung function decline at one year in a heterogeneous ILD population. We found that 37% of the patients had progression over one year, which is consistent with previous observations^29,30^. By using multi-step regression analysis, we could show that that serum KL-6, as a continuous variable or by strata, was the most consistent predictor of disease progression at one year (odds ratios between 1.3 and 1.9 for all ILD groups, Supplement Table 2). The sensitivity analyses confirmed a good correlation of the KL-6 strata with Dlco and FVC at baseline and with the decline in FVC, but not Dlco, at one year. The use of strata allowed us to obtain an incremental risk of disease progression and a score with a better performance compared to clinical parameters or KL-6 alone. In a previous monocentric study in 205 patients with fibrotic ILD^31^, serum KL-6 strata were significant predictors of progression and were included in a simple score (GK score) to discriminate patients at high and low risk to develop disease progression at any time. The serum KL-6 strata, identified by ROC analysis, were similar to those from our study. Compared to the present study, Jehn et al. investigated only fibrotic ILDs (IPF and NSIP), leaving a possible application of the GK score in nonfibrotic ILD unexplored. We found that the performance of the P-ILD score to predict disease progression at one year was not homogeneous across all ILD subtypes, with the highest value observed in IIP and the lowest in HP patients (Fig. 4). The sensitivity analyses (Supplement) using different definitions of progression confirmed this finding. We do not have a single explanation for this difference. On one side, it could be related to the high variability of serum KL-6 concentrations in patients with HP, as recently pointed out by a meta-analysis^32^. On the other side, since disease progression and prognosis of HP depend on antigen identification , contact exposure or avoidance can impact the disease course^33,34^. Although the rate of progression at one year in HP patients (31%) was similar to the other ILD groups, we cannot exclude that the antigen exposure, not included among the predictors, could have a higher weight than the 5 variables included in the P-ILD score. This intriguing aspect needs further investigation.

Our score does not include HRCT pattern or the fibrosis quantitative score as predictors of progression at one year. It is known that UIP pattern is one of the strongest mortality predictors across ILD^35,36^, but the association between UIP pattern and rate of FVC decline in ILD other than IPF has not been completely elucidated^36,37^. We did not find a significant correlation between UIP pattern or fibrosis score at baseline and progression at one year (supplement Table 1). Moreover, no correlation between UIP pattern and serum KL-6 concentrations was observed. This is somehow in contrast with previous studies which showed a good correlation between serum KL-6, UIP pattern and fibrosis extent at HRCT^38–40^, mostly in CTD-ILD^41–44^. A possible explanation can be related to the timeframe to define ILD progression. Due to data availability, we used twelve months, which is a short period to define progression in diseases like CTD-ILD or HP. Other progression criteria, for example PF-ILD^45,46^, consider two years as a reasonable time to catch a progression signal. Interestingly, as we compared patients with higher vs. lower risk of progression based on the P-ILD score (cut off at 5 points), UIP pattern and a fibrosis score ≥ 10% were significantly more frequent in the high-risk group (supplement Table 4), confirming the validity of the score.

A further strength of the P-ILD score including KL-6 concentrations at baseline is that clinical characteristics of patients at high or low risk of progression can be separated more effectively than by using each variable alone (supplement Table 4). This is in line with previous studies, which have tried to improve the GAP index by adding baseline serum KL-6^31,47^.

Despite the novel findings, our study has several limitations. First, we included patients from 6 centers, but we were not able to identify a derivation and validation cohort, due to the heterogeneity of the patient’s population. Second, we did not collect data on comorbidities or co-medications at baseline, variables that can influence development of progression and even the serum concentration of KL-6. In addition, we cannot exclude that KL-6 serum concentrations strata can slightly vary in other study populations due to different ethnicity or heterogeneity of included ILD^48,49^. Finally, data on acute exacerbations, a complication known to accelerate disease progression, were not available for all the centers and could not be included in the analysis.

## Conclusion

In conclusion, our study shows that baseline serum KL-6 concentrations, included in a risk score with other clinical and functional variables, may help to better stratify patients for the risk of disease progression at one year, compared to any individual predictor.

## Supplementary Information

Below is the link to the electronic supplementary material.

Supplementary Material 1

## Data Availability

The data that support the findings of this study are not openly available due to reasons of sensitivity and are available from the corresponding author upon reasonable request.

## Abbreviations

AUC: Area under the ROC curve
BMI: Body mass index
CTD/IPAF-ILD: Connective tissue disease or autoimmune feature-ILD
DLco: Diffusing capacity of the lung for carbon monoxide
FVC: Forced vital capacity
GAP index: Gender, age, physiology index
HRCT: High resolution computed tomography
HP: Chronic hypersensitivity pneumonitis
ILD: Interstitial lung disease
IIP: Idiopathic interstitial pneumonia
IPF: Idiopathic pulmonary fibrosis
KL-6: Krebs von den Lungen-6
% pred: Percentages of predicted normal values
PFT: Lung function test
PP: Progressive phenotype
ROC: Receiver operating curve
SD: Standard deviation
IQR: Interquartile range
