# Supplementary material for: Serum KL-6 as a biomarker to predict progression at one year in interstitial lung disease
Source: Sci Rep. 2025 Oct 9;15:35243. doi: 10.1038/s41598-025-22483-4 (PMC12511299; doi:10.1038/s41598-025-22483-4)
Supplement: Supplementary file 1 — Supplementary Material 1 [file 41598_2025_22483_MOESM1_ESM.docx]

**Supplemental Material VAMOS manuscript**

**APPENDIX A: STATISTIC METHODS, TABLES AND FIGURES**

**Statistic methods: additional details on logistic regression**

Logistic regression analysis with a stepwise selection was performed for qualitative and quantitative variables based on the contribution of each variable defined by the Chi-square (Log ratio) and the probability (Pr >LR). The Type II analysis and the odds ratios (exponent of standardized β coefficient) from logistic regression was used to assess the contribution of each variable, overall and by ILD category. Due to the weight heterogeneity according to ILD category, we used the Hosmer–Lemeshow test to evaluate agreement between the probability predicted by Logit model and observed proportions of events (progression vs. stable at 1-year). Small values with large p-values indicate a good fit to the data while large values with p-values below 0.05 indicate a poor fit.

The selected variables were stratified by analyzing different threshold values based on the sum of sensitivity + specificity and the accuracy determined by receiver operating characteristic (ROC) curve analysis.

The odds ratios (OR) were calculated with the exponent of model regression coefficients.

**Supplement Table 1** Logistic regression analysis for selecting qualitative variables associated with progression at 1 year in the entire cohort.

| **Variable** | **DF** | **Chi² (LR)** | **Pr > LR** |
| --- | --- | --- | --- |
| **Gender** (F/M) | 1 | 5.138 | **0.023** |
| **ILD subtype** | 2 | 0.598 | 0.742 |
| **UIP pattern at HRCT** (Yes/No) | 1 | 1.727 | 0.189 |
| **Fibrosis score** (</≥10%) | 1 | 0.045 | 0.833 |
| **Emphysema Extent** (Yes/No) | 1 | 0.566 | 0.452 |
| **Dyspnea** (Yes/No) | 1 | 0.171 | 0.679 |
| **GAP stage** | 2 | 1.065 | 0.587 |
| Abbreviations: Chi², Chi-square; LR, Likelihood Log ratio; DF, degrees of freedom. | | | |

**Supplement Table 2.** Logistic regression for predictors of disease progression at one year according to underlying ILD.

| Category | **all ILDs** | | | **IIP (43%)** | | | **CTD/IPAF (27%)** | | | **HP/cHP (30%)** | | |
| --- | --- | --- | --- | --- | --- | --- | --- | --- | --- | --- | --- | --- |
| Source | Chi² (LR) | Pr > LR | OR (expβ) | Ch² (LR) | Pr > LR | OR (expβ) | Ch² (LR) | Pr > LR | OR (expβ) | Ch² (LR) | Pr > LR | OR (expβ) |
| Age (years) | 0.285 | 0.594 | 1.088 | 1.383 | 0.240 | 1.353 | 1.282 | 0.258 | 1.457 | 1.311 | 0.252 | 0.727 |
| BMI (kg/m²) | 0.320 | 0.572 | 1.092 | 0.011 | 0.917 | 1.026 | 2.922 | 0.087 | 1.753 | 0.127 | 0.721 | 0.902 |
| B-KL6 (U/mL) | 6.579 | **0.010** | 1.481 | 6.919 | **0.009** | 1.898 | 1.874 | 0.171 | 1.560 | 0.843 | 0.359 | 1.300 |
| B-FVC, %pred | 0.008 | 0.928 | 1.014 | 1.211 | 0.271 | 0.756 | 3.027 | 0.082 | 1.811 | 0.149 | 0.699 | 0.893 |
| Gender (F/M) | 4.502 | **0.034** | 1.393 | 4.820 | **0.028** | 1.741 | 2.103 | 0.147 | 1.624 | 0.110 | 0.741 | 1.099 |
| Statistic | Chi² | Pr > Chi² | | Chi² | Pr > Chi² | | Chi² | Pr > Chi² | | Chi² | Pr > Chi² | |
| -2 Log (Likelihood) | 11.272 | **0.046** | | 13.089 | **0.023** | | 7.842 | 0.165 | | 2.718 | 0.743 | |
| Hosmer-Lemeshow | 3.738 | 0.928 | | 11.091 | 0.270 | | 10.757 | 0.293 | | 3.341 | 0.911 | |
| Significance is indicated by bold values.  Type II analysis for Chi-square (LR) – OR calculated as exponential of standardized β-coefficient.  Hosmer-Lemeshow test: large values with p-values below 0.05 indicate a poor fit.  Abb.: BMI = body mass index; B-FVC = baseline forced vital capacity; B-KL-6 = baseline Krebs von den Lungen-6; Chi², Chi-square; LR= Log ratio;  OR = odds ratio | | | | | | | | | | | | |

**Supplement Figure 1** ROC curves analysis of selected quantitative variables for prediction of disease progression at one year.


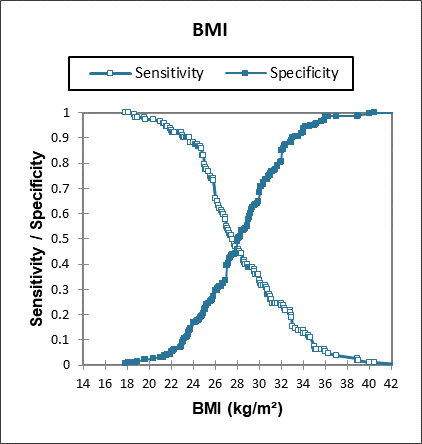

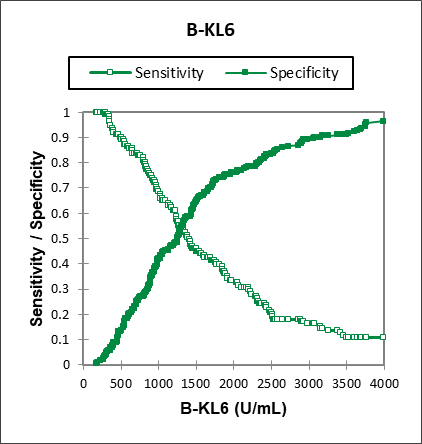

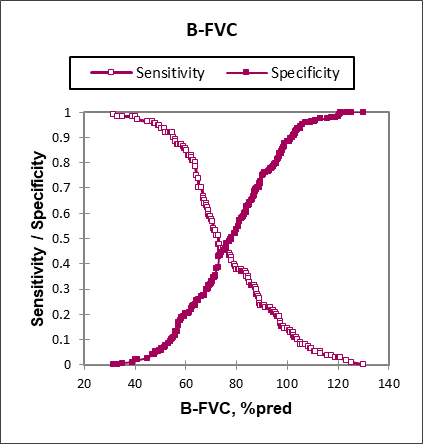

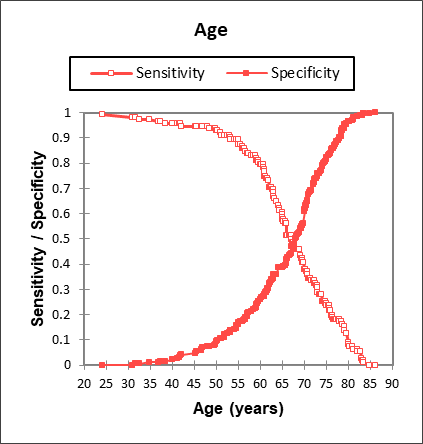


**Supplement Figure 2.** Contingency (adjusted residuals) for progressive strata of quantitative variable selected by logistic regression analysis.

**Supplement Table 3** Final prediction’s model with selected variable strata for predictors for progression at 1 year.

| **Variable** | **DF** | **Chi² (LR)** | **Pr > LR** |
| --- | --- | --- | --- |
| **Gender-M** | 1 | 4.463 | 0.035 |
| **Age** <60; 60-75; >75 | 2 | 3.040 | 0.219 |
| **BMI** <22; 22-32; >32 | 2 | 5.937 | 0.051 |
| **KL-6** <750; 750-1750; >1750 | 2 | 5.927 | 0.052 |
| **FVC** >70; ≤70 | 1 | 2.278 | 0.131 |
| **Statistic** | DF | Chi² | Pr > Chi² |
| -2 Log(Likelihood) | 8 | 21.163 | 0.007 |
| Hosmer-Lemeshow | 7 | 1.333 | 0.988 |
| Abbreviations: Chi², Chi-square; LR, Likelihood Log ratio; DF, degrees of freedom | | | |

**Supplement Figure 3**: Logistic regression standardized coefficients of selected variable (A) and ROC aera under curve analysis (B).


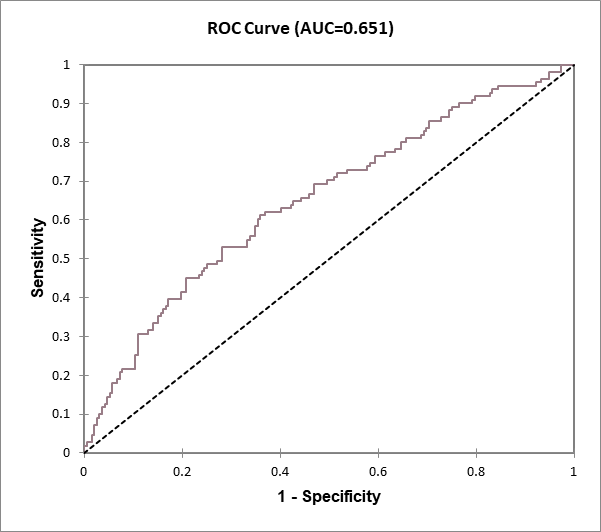
A B

Event 1 =Decline at 1y

**Supplement Table 4.** Baseline characteristics of patients according to P-ILD risk score for disease progression at one year.

|  | **All subjects** | **P-ILD HR** | **P-ILD LR** | ***p value*** |
| --- | --- | --- | --- | --- |
|  | **N= 303** | **N=112 (37%)** | **N=191 (63%)** |  |
| **Age**, y (IQR) | 68.0 (60.8-73.8) | 69.3 (63.1-74.9) | 66.6 (57.1-73.0) | **0.004** |
| **BMI**, kg/m² (IQR) | 28.0 (25.6-31.0) | 28.5 (26.0- 32.9) | 28.0 (25.2-30.1) | **0.011** |
| **Male gender**, n (%) | 182 (60.1) | 91 (81.3) | 91 (47.6) | **<0.0001** |
| **KL-6**, U/mL (IQR) | 1287 (819-2177) | 2125 (1300-2998) | 986 (576-1441) | **<0.0001** |
| **FVC**, %pred (IQR) | 76.0 (64.0-90.0) | 67.1 (58.8-79.3) | 82.2 (72.0-95.6) | **<0.0001** |
| **DLco**, %pred (IQR) | 54.0 (41.9-68.7) | 45.0 (37.0-60.1) | 57.1 (45.0-72.0) | **<0.0001** |
| **GAP stages**, I/II/III (%) | 60.4/33.8/5.8 | 30.9/56.4/12.7 | 78.1/20.2/1.6 | **<0.0001** |
| **Emphysema**, Yes (%) | 43 (16.6) | 18 (18.0) | 25 (15.7) | 0.838 |
| **Dyspnea**, Yes (%) | 222 (73.3) | 91 (81.3) | 131 (68.6) | 0.108 |
| **Fibrosis score**,  </≥10% (%) | 28.3 / 71.7 | 10.1 / 89.9 | 39.2 / 60.8 | **<0.0001** |
| **UIP pattern at HRCT**, | 48.8 / 51.2 | 65.3 / 34.7 | 38.4 / 61.6 | **<0.0001** |
| likely/unlikely (%) |  |  |  |  |
| **ILD categories**, n (%) |  |  |  | **0.0003** |
| CTD-ILD/IPAF | 82 (27.1) | 20 (17.9) | 62 (32.5) | **0.007** |
| HP | 90 (29.7) | 27 (24.1) | 63 (33.0) | 0.119 |
| IIP (incl. IPF) | 131 (43.2) | 65 (58.0) | 66 (34.6) | **0.0001** |
| **FVC %pred decline**  in % (IQR) | 0% (-8% to 7%) | -3% (-10% to 5%) | 1% (-7% to 8%) | **0.020** |
| **DLCO %pred decline**  in % (IQR) | -4% (-14% to 7%) | -13% (-21% to 3%) | -2% (-10% to 8%) | **0.0001** |
| **Progressors (per PFT), n (%)** | 111 (36.6) | 61 (54.5) | 50 (26.2) | **<0.0001** |
| Abbreviations: BMI = body mass index; y = years; PFT = lung function test; FVC = forced vital capacity; DLco = diffusing capacity for carbon monoxide; GAP = gender, age, physiology; HR=high risk; LR= low risk; ILD = interstitial lung disease; IIP = idiopathic interstitial pneumonia; CTD/IPAF-ILD = connective tissue disease or autoimmune feature-ILD; HP = hypersensitivity pneumonitis; KL-6 = Krebs von den Lungen-6; P-ILD = progression of ILD; IQR = interquartile range; n = number. | | | | |

**APPENDIX B: SENSITIVITY ANALYSES**

**Sensitivity analysis strategies**

The primary analysis and the proposed model (P-ILD) was based on the following disease progression definition, ie. relative decline ≥10% in FVC %pred or ≥15% in Dlco %pred (Khanna D, et al J Rheumatol. 2015 Nov;42(11):2168-71.).

A P-ILD score >5 points was considered at high-risk (HR) of progression within 12 months. The analysis by sub-groups of ILDs showed a lack of performance for HP group.

We performed post hoc analyses to compare

1. the proportion of progressors and stable cases depending on the progression definition
2. the distribution of HR and LR defined using the P-ILD score model by different progression definitions:
   1. by ILD categories
   2. Overall but excluding HP group, on which the application of the P-ILD risk model did not reach a significant level of performance

**Analysis 1: Proportion of progressors and stable cases depending the progression definition**

- 1. Applying PPF definition
- PPF definition (Raghu et al , Am J Respir Crit Care Med 2022; 205(9): 18-47.): absolute decline in FVC of ≥5 or absolute decline in DLCO of ≥10 within one year of follow-up.

| **Summary across groups:** | | | |  |
| --- | --- | --- | --- | --- |
|  |  |  | |  |
| Contingency table (Progressor vs Stable relative decline criteria) \ PPF criteria | | | | |
|  |  | |  |  |
| **ILD cat.** | **Progressor vs Stable relative decline criteria) \ PPF** | | **PPF-P** | **PPF-S** |
| CTD/IPAF | P | | 21 | 4 |
|  | S | | 6 | 51 |
| HP | P | | 33 | 1 |
|  | S | | 10 | 46 |
| IIP | P | | 41 | 11 |
|  | S | | 3 | 76 |

Abbr.: P= progressor, S= stable

Graphical representation

- Results: 36.6% (111/303) progressors (P-ILD) by using relative physiologic FVC decline *vs.* 37.6% (114/303) progressors by using PPF definition

1.2 Applying Meaningful Progression (MP) definition

- Meaningful Progression (MP) definition by Distler et al (Predictors of progression in systemic sclerosis patients with interstitial lung disease. Eur Respir J. 2020 May 14;55(5):1902026.): either a decline in forced vital capacity (FVC) from baseline of ≥10%, or a decline in FVC of 5-9% in association with a decline in DLco of ≥15% represents progression.

| Contingency table | | | |
| --- | --- | --- | --- |
|  |  |  |  |
| **ILD cat.** | **Progressor vs Stable relative decline criteria) \ MP** | **MP-P** | **MP-S** |
| CTD/IPAF | P | 18 | 7 |
|  | S | 0 | 57 |
| HP | P | 25 | 9 |
|  | S | 0 | 56 |
| IIP | P | 35 | 17 |
|  | S | 0 | 79 |

Abbr.: P= progressor, S= stable

Graphical representation

- Results: 36.6% (111/303) progressors (P-ILD) by using relative physiologic FVC decline *vs.* 25.7% (78/303) progressors by MP definition (MP-P)

**Analysis 2: Performance of the P-ILD score according to the different disease progression definitions as per Analysis 1**

2.1 Performance of the P-ILD score if PPF definition of progression is applied

|  | | |  |
| --- | --- | --- | --- |
|  |  |  |  |
| Contingency table (P-ILD risk>5 \ PPF criteria (aFVC>5 OR aDlco>10)): | | | |
|  |  |  |  |
| **ILD cat.** | **P-ILD risk >5 \ PPF criteria** | **PPF-P** | **PPF-S** |
| CTD/IPAF | HR | 9 | 11 |
|  | LR | 18 | 44 |
| HP | HR | 13 | 14 |
|  | LR | 30 | 33 |
| IIP | HR | 29 | 36 |
|  | LR | 15 | 51 |

Abbr.: P= progressor, S= stable

Graphical representation

2.2. Performance of the P-ILD score if MP definition of progression is applied

| Contingency table (P-ILD risk >5 \ Meaningful Progression (MP) Distler et al (2020)): | | | | | | |
| --- | --- | --- | --- | --- | --- | --- |
|  |  |  |  | | | |
| **ILD cat.** | **P-ILD risk \ MP strata** | | | **MP-P** | **MP-S** |  |
| CTD/IPAF | HR | | | 8 | 12 |  |
|  | LR | | | 10 | 52 |  |
| HP | HR | | | 7 | 20 |  |
|  | LR | | | 18 | 45 |  |
| IIP | HR | | | 23 | 42 |  |
|  | LR | | | 12 | 54 |  |

Abbr.: P= progressor, S= stable

Graphical representation

**Summary of the Analysis 1 and 2**

Using different progression definitions:

- Performance of P-ILD score in HP group did not improve
- Performance of P-ILD score maintained for IIP, but not for CTD/IPAF using PPF criteria
- Performance of P-ILD score maintained with similar accuracy for IIP and CTD/IPF by using MP definition

**Conclusion regarding Analysis 1 and 2:**

P-ILD model and score aligns more with other common models using relative decline of lung function parameters (OMERACT, MP or PF-ILD model) than those using absolute decline (PPF). This sensitivity analysis is therefore supportive for our model.

**Analysis 3: Performance of P-ILD score according to different criteria of parameter strata** (ILD included: IIP, CTD/IPAF).

Methods: We re-analyzed the overall performance after excluding HP on which the application of the risk model did not reach a significant level of performance and compared the overall link between the dichotomization of ILD –P scoring in HR or LR and different definitions or parameters. Same statistical methods as per primary analysis.

- 1. **Predictive value of LR for stable outcome within a year remains high and comparable across the different parameter strata and progression definitions**
- 78.1% LR had a stable outcome (vs progression definition reference)
- 86.7% LR had B-KL6 <1750 U/mL
- 83.6% LR had relative decline <10% in FVC % pred
- 74.2% LR had PPF stable outcome
- 82.8% LR had MP stable pattern

**3.2 Predictive value of HR for progressor outcome within a year showed variability across the different parameter strata and progression definitions**

- 57.6% HR had a progressive outcome (vs progression definition reference)
- 60.0% HR had B-KL6 >1750 U/mL
- 28.2% HR had relative decline ≥10% in FVC %pred
- 44.7% HR had PPF progressor outcome
- 36.5% HR had MP progressor outcome

Graphical representation of Analysis 3 results:

Original Risk score performance

Applying PPF criteria

Applying MP criteria

**Conclusion regarding analysis 3:** P-ILD risk score (for IIP and CTD/IPAF) is associated to progression/stable status according to different criteria with significant p-value: The chi square values (Chi²) were 28.32 (p<0.0001) for reference criteria, 61.55 (p<0.0001) for B-KL6 strata, 8.23 (p=0.005) for PPF criteria 10.16 (p=0.0001) for MP criteria, and 4.29 (p<0.038) for relative decline in FVC %pred only.

**Analysis 4: Linear regression for progression at 1 year based on a different definition (PPF).**

We performed a linear regression for progression at one year based on the PPF definition, analysing the correlation with baseline values of KL-6 (B-KL-6) and DLco (B-DLCO) % predicted.

**Conclusion regarding Analysis 4:** the absolute decline at 1 year in FVC and Dlco depends on the baseline value of the respective parameters, revealing a weakness of PPF criteria in this clinical setting, and corroborating the use of the progression definition based on relative decline used in our primary analysis.

**Analysis 5: investigation of the link between KL-6 strata included in the P-ILD risk score model and baseline FVC and DLco.**

Method: First, we compared the distribution of baseline FVC (B-FVC) and DLco values (B-DLco) according to the KL-6 strata, in order to verify whether a correlation/ link exists. Then, we compared the relative decline (%) of FVC and Dlco at one year according to the KL-6 strata.

4.1. Summary statistics

4.2. Distribution of baseline FVC values and KL6 strata

4.3. Distribution of baseline DLco values and KL6 strata

4.4 Distribution of relative decline (%) of FVC at 1 year according to KL6 strata (at baseline)

4.5 Distribution of relative decline (%) of DLco at 1 year according to KL6 strata (at baseline)

**Conclusions regarding Analysis 5:**

- the results corroborate the inclusion of these KL-6 strata in the risk P-ILD score, due to the significant correlation with baseline lung function tests and with relative decline in FVC at 1-year.
